# Wide-Range Measurement of Thermal Preference—A Novel Method for Detecting Analgesics Reducing Thermally-Evoked Pain in Mice

**DOI:** 10.3390/molecules26030612

**Published:** 2021-01-25

**Authors:** Kinga Sałat, Anna Furgała-Wojas, Michał Awtoniuk, Robert Sałat

**Affiliations:** 1 Department of Pharmacodynamics, Chair of Pharmacodynamics, Jagiellonian University Medical College, 9 Medyczna St., 30-688 Krakow, Poland; anna.furgala@student.uj.edu.pl; 2 Institute of Mechanical Engineering, Warsaw University of Life Sciences, 164 Nowoursynowska St., 02-787 Warsaw, Poland; michal_awtoniuk@sggw.edu.pl; 3 Faculty of Electrical and Computer Engineering, Cracow University of Technology, 24 Warszawska St., 31-155 Krakow, Poland; robert.salat@pk.edu.pl

**Keywords:** oxaliplatin, thermal preference, deep learning, image analysis, control, duloxetine, pregabalin, mice

## Abstract

Background: Wide use of oxaliplatin as an antitumor drug is limited by severe neuropathy with pharmacoresistant cold hypersensitivity as the main symptom. Novel analgesics to attenuate cold hyperalgesia and new methods to detect drug candidates are needed. Methods: We developed a method to study thermal preference of oxaliplatin-treated mice and assessed analgesic activity of intraperitoneal duloxetine and pregabalin used at 30 mg/kg. A prototype analgesiameter and a broad range of temperatures (0–45 °C) were used. Advanced methods of image analysis (deep learning and machine learning) enabled us to determine the effectiveness of analgesics. The loss or reversal of thermal preference of oxaliplatin-treated mice was a measure of analgesia. Results: Duloxetine selectively attenuated cold-induced pain at temperatures between 0 and 10 °C. Pregabalin-treated mice showed preference towards a colder plate of the two used at temperatures between 0 and 45 °C. Conclusion: Unlike duloxetine, pregabalin was not selective for temperatures below thermal preferendum. It influenced pain sensation at a much wider range of temperatures applied. Therefore, for the attenuation of cold hypersensitivity duloxetine seems to be a better than pregabalin therapeutic option. We propose wide-range measurements of thermal preference as a novel method for the assessment of analgesic activity in mice.

## 1. Introduction

Oxaliplatin (trans-l-diaminocyclohexane oxalate platinum (II)) belongs to third-generation platinum-based chemotherapeutic agents. This drug has been placed on the World Health Organization’s List of Essential Medicines. As other platinum-based compounds (e.g., cisplatin and carboplatin), oxaliplatin impairs the proliferation of tumor cells by forming deoxyribonucleic acid-platinum adducts and this activity results in cancer cell death. In humans, oxaliplatin is used in combination with folinic acid and 5-fluorouracil (FOLFOX therapy) or with folinic acid, 5-fluorouracil and irinotecan (FOLFOXIRI therapy) as the first-line or adjuvant treatment of colorectal cancer [1].

Chemotherapy-induced peripheral neuropathy (CIPN) is the main limitation of using oxaliplatin to treat cancer. CIPN comprises acute, transient neuropathy that is observed in almost 90% of patients within hours after oxaliplatin infusion, and a chronic form that occurs in 70% of oxaliplatin-treated patients. Importantly, these delayed complications might be persistent and are likely to be progressively aggravated [2]. Several potential mechanisms have been taken into consideration to establish phenomena underlying CIPN caused by oxaliplatin and currently it is thought that a rapid and non-enzymatic transformation of this drug to metabolites, such as oxalate, monochloro-, dichloro- and diaquodiaminocyclohexane platinum derivatives, is responsible for neurotoxicity of oxaliplatin. This metabolic pathway occurs in the blood plasma and it consists in the replacement of the oxalate moiety with chloride ions and water. The formation of these metabolites is a unique feature of oxaliplatin and it distinguishes this drug from other platinum-based compounds used in the treatment of cancer. Of note, oxalate that is formed from oxaliplatin is responsible for the observed cold-induced hypersensitivity in patients on oxaliplatin therapy. Oxalate acts as a calcium chelator, which stimulates the removal of extracellular calcium ions, and this leads to the increased sodium conductance, neuronal depolarization and hyperexcitability [1].

In contrast to tactile allodynia, which can be effectively attenuated by analgesic adjuvants (e.g., pregabalin), hypersensitivity to cold stimuli is regarded as the main and still not addressed clinical issue in patients on oxaliplatin therapy. This clinical entity is pharmacoresistant to available analgesics and according to the guidelines of the American Society of Clinical Oncology (ASCO) only duloxetine, an analgesic adjuvant is moderately recommended for the prevention of oxaliplatin-induced cold-exacerbated pain [1]. Considering this, novel treatment options and novel analgesics for the alleviation of cold allodynia/hyperalgesia are strongly needed.

Animal (preclinical) models of CIPN are particularly useful in the search for analgesics relieving CIPN-related pain as both forms of CIPN observed in patients are also noted in mice and rats treated with oxaliplatin. In laboratory animals these acute reactions usually occur within 2–3 h after oxaliplatin injection, while the subacute responses are noted several days later [3]. Moreover, it has been shown that the acute painful symptoms detected as early as 2 h after oxaliplatin injection might be different from those that appear 24 h to 7 days later [3]. For instance, using a rat model of CIPN, it has been demonstrated that cold allodynia and cold hyperalgesia develop earlier than mechanical allodynia [3].

In recent years, many studies have been focused on the assessment of the optimal ambient temperature for laboratory mice and other species. There has been a growing interest in such research because laboratory animal welfare is now regarded as a key issue in the in vivo studies. Research focused on studying behavioral thermoregulatory responses has shown that non-neuropathic mice generally prefer an environment maintained at 22–30 °C, while temperatures exceeding 34 °C might be recognized as heat-stress [4]. In a rat study that used a Peltier-cooled cold plate [5], it has been shown that in naïve rats the threshold for eliciting cold pain behavior is 5 °C, while temperatures 10–25 °C could be considered innocuous for non-neuropathic subjects. In this study, for neuropathic animals the withdrawal threshold for cold allodynia was established at 15 °C. The results of this research have shown that the thermal preferendum—an essential primitive thermoregulatory response of many animal species, which serves to select a comfortable thermal environment—differs significantly between non-neuropathic and neuropathic animals.

As mentioned above, pain management strategies have failed to alleviate cold-exacerbated pain caused by oxaliplatin. On the other hand, it is also likely that the in vivo methods used at the preclinical stage (i.e., behavioral tests) might not be sensitive enough to detect analgesic active compounds, or the parameters used to assess analgesic activity (i.e., temperatures at which chemical compounds are tested) are suboptimal. In the only available study focused on measuring thermal preference of animals treated with oxaliplatin, Balayssac and colleagues [6] have demonstrated thermal hypersensitivity at 12, 20 and 35 °C in oxaliplatin-treated rats. In their study the reference drug-duloxetine (2.5 mg/kg, intraperitoneal administration) reversed oxaliplatin-induced cold hypersensitivity at 20 °C. Importantly, the cold plate test or the water immersion test, which are widely applied to test drugs and potential drug candidates for the treatment of oxaliplatin-induced cold hyperalgesia use much lower temperatures, so there is a potential risk that antiallodynic/antihyperalgesic properties of test compounds in the animal model of CIPN caused by oxaliplatin are assessed at temperatures that are not optimal for this model.

Considering this, in order to develop a sensitive and accurate method to study the thermal preference of mice with symptoms of oxaliplatin-induced cold hypersensitivity and to assess the activity of analgesics in this model, we undertook this preliminary research to compare the thermal preference of naive mice and mice treated with oxaliplatin, pregabalin and duloxetine in a broad range of temperatures (0–45 °C). We used duloxetine and pregabalin to compare their effect on the thermal nociceptive threshold of mice. These two drugs are widely used to attenuate CIPN-related neuropathic pain symptoms in humans but it is suggested that they might have a distinct efficacy in this clinical condition, with duloxetine being more effective than pregabalin in reducing oxaliplatin-induced cold hypersensitivity. Hence, by studying thermal preference of neuropathic, oxaliplatin-treated mice exposed to pregabalin or duloxetine, we aimed to compare their effect on the thermal pain threshold at a wide range of temperatures applied. For this purpose we used a prototype analgesiameter designed and constructed by our research team. In this device the current temperature in each zone is controlled, which is an innovative solution. This enables a very precise examination of pain sensitivity of mice. By recording the image with the use of a very sensitive camera and by using advanced methods of image analysis (deep learning and machine learning), we determined thermal preferences of animals. Therefore, we not only assessed the effect of oxaliplatin on pain threshold in mice and determined the analgesic effectiveness of drugs tested but we also propose wide-range measurements of thermal preference as a novel method for the assessment of analgesic activity of drugs and drug candidates in animal models that are based on measuring thermal hypersensitivity.

## 2. Results

### Effect of Duloxetine and Pregabalin on Thermal Preference in Mice

**Figure 1:**
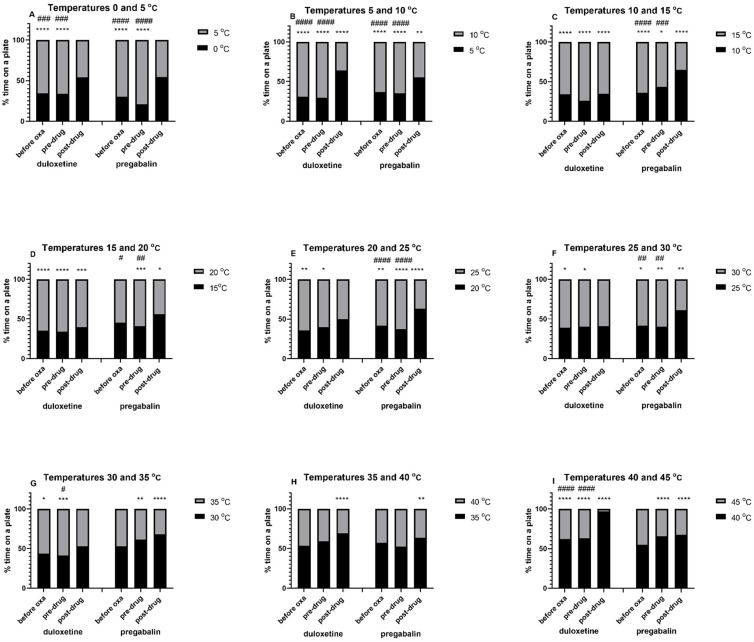
Effect of oxaliplatin, duloxetine and pregabalin on the thermal place preference measured in mice. The two-plate thermal place preference test was conducted at various temperature ranges between 0 and 45 °C. Results are shown as % time spent in a particular thermal zone measured at 3 distinct time points: before oxaliplatin injection (“before oxa”), 3 h after the injection of oxaliplatin (“pre-drug”) and 1 h after intraperitoneal injection of duloxetine or pregabalin, each used at the dose of 30 mg/kg (“post-drug”). Statistical analysis: repeated measures ANOVA and Tukey’s multiple comparison significance vs. time (s) spent on the second plate at the same time point of testing: * *p* < 0.05, ** *p* < 0.01, *** *p* < 0.001 and **** *p* < 0.0001; significance vs. time (s) spent on the same plate after duloxetine, or pregabalin administration: # *p* < 0.05, ## *p* < 0.01, ### *p* < 0.001 and #### *p* < 0.0001. Oxa: oxaliplatin; *n* = 8–10.

As shown in Figure 1A–G, when exposed to two distinct temperatures, mice before duloxetine administration showed a statistically significant (*p* < 0.05) preference for zones with a higher temperature set. This effect was noted for temperatures between 0 and 35 °C and was particularly strong at temperatures between 0 and 15 °C. Similarly, before pregabalin administration statistically significant (*p* < 0.05) preference towards a plate set at a higher temperature was shown at temperatures between 0 and 30 °C. Of note, in these mice, at temperatures 15–20 °C (Figure 1D) and 30–35 °C (Figure 1G), before oxaliplatin treatment the % time spent on one of the plates did not differ significantly from that spent on the second one. In contrast to this, the injection of oxaliplatin to these mice significantly (*p* < 0.01) increased % time spent on one of the two plates used (a warmer one—Figure 1D, or a colder one—Figure 1G).

Before duloxetine and oxaliplatin administration the lower temperature of the two used was preferred only in experiments at temperatures 35 °C and above (Figure 1H,I). In these mice, a statistically significant (*p* < 0.0001) preference towards a plate set at a lower temperature was noted in the experiment using temperatures 40 °C and 45 °C (Figure 1I). Before the administration of pregabalin and oxaliplatin a slight (statistically not significant) preference towards a plate with a lower temperature was observed in experiments involving temperatures: 30–35 °C (Figure 1G), 35–40 °C (Figure 1H) and 40–45 °C (Figure 1I). In these mice, oxaliplatin caused a highly significant (*p* < 0.01) preference towards a colder plate of the two used in the experiment involving 30–35 °C (Figure 1G) and 40–45 °C (Figure 1I).

Interestingly, duloxetine (30 mg/kg, i.p.) caused thermal preference loss at some temperature ranges: 0–5 °C (Figure 1A), 20–25 °C (Figure 1E) and 30–35 °C (Figure 1G). This loss of thermal preference after duloxetine injection may be regarded as an indicator of its analgesic effect and points out for thermal ranges at which this activity can be detected. It had no effect on the thermal preference at 25–30 °C (Figure 1F). For the pregabalin-treated group thermal preference was lost only for 0–5 °C (Figure 1A).

A statistically significant thermal preference for the plate set a higher temperature was maintained after duloxetine treatment for temperatures: 10–15 °C (*p* < 0.0001, Figure 1C) and 15–20 °C (*p* < 0.001, Figure 1D), likely showing that at these temperature ranges the analgesic activity of duloxetine was not detected. In turn, after duloxetine administration thermal preference was reversed towards lower temperatures for 5–10 °C (*p* < 0.0001, Figure 1B), 35–40 °C (*p* < 0.0001, Figure 1H) and 40–45 °C (*p* < 0.0001, Figure 1I). This might also suggest that (i) at 5–10 °C duloxetine elevates the pain threshold of oxaliplatin-treated mice and (ii) at 35–40 °C and 40–45 °C (Figure 1H,I, respectively) heat stress avoidance is observed, so the animals choose thermal zones closer to their thermal preferendum.

In neuropathic, oxaliplatin-treated mice, thermal preference was lost after pregabalin administration in the experiment involving temperatures 0 °C and 5 °C (Figure 1A). Compared to predrug measurements, pregabalin reversed thermal preference of neuropathic mice for plates set at 5–10 °C (significant at *p* < 0.01, Figure 1B), 10–15 °C (*p* < 0. 0001, Figure 1D), 15–20 °C (*p* < 0.05, Figure 1D), 20–25 °C (*p* < 0.0001, Figure 1E) and 25–30 °C (*p* < 0.01, Figure 1F). The preference of pregabalin-treated mice towards a plate set at a lower temperature was maintained during the experiment using plates set at 30–35 °C (*p* < 0.0001, Figure 1G), 35–40 °C (*p* < 0.01, Figure 1H) and 40–45 °C (*p* < 0.0001, Figure 1I).

Taken together, our preliminary study indicated a potential analgesic effect of duloxetine and pregabalin measured as a loss of preference for a zone with a higher temperature, or as a reversal of preference towards a zone with a lower temperature of the plate. Importantly, our research enabled us to differentiate between the mode of analgesic action of the two drugs tested, i.e., duloxetine and pregabalin. Considering that increased sensitivity to cold is a key symptom of oxaliplatin-induced CIPN, pain management strategies for this debilitating clinical condition should be focused on the attenuation of cold hypersensitivity, i.e., drugs should provide analgesia selectively at low temperatures without impairing nociception at temperatures within thermal preferendum.

We also compared the degree of thermal preference induced by both drugs. This analysis showed that before the administration of oxaliplatin, duloxetine or pregabalin to mice, the % time spent by the animals was similar in both cohorts of animals (i.e., duloxetine group and pregabalin group) and at temperatures 0–30 °C the preference towards a colder plate of the two used was below 50% (Figure 1A–F). At temperatures above 35 °C, the thermal preference towards a colder plate was also similar in these groups but at these temperature ranges it was higher than 50% (Figure 1H,I). Interestingly, the analysis of % time spent in the two thermal zones before oxaliplatin treatment showed however the opposite results obtained for both control groups in the experiment set at 30–35 °C (% time spent on a colder plate in both control groups: 44% vs. 53%, Figure 1G). These apparently opposite results can be explained as follows: the temperature range between 30 and 35 °C is the upper limit of the temperature range within the mouse thermal preferendum (below heat stress), hence the observed differences do not seem to result from method flaws or model abnormalities, but they might reflect a natural variation in the thermal sensitivity of individuals observed in general population.

Additionally, in oxaliplatin-treated cohorts the degree of thermal preference was similar between 0 and 30 °C (preference for the colder plate was below 45%, Figure 1 A–F) and between 35 and 45 °C (preference for the colder plate was above 50%, Figure 1 H,I), with apparently opposite results for 30–35 °C (41% vs. 61% in preduloxetine and prepregabalin groups, respectively, Figure 1G).

In each cohort of animals we also compared predrug (after oxaliplatin administration) and postdrug (after duloxetine or pregabalin administration) effects of treatment on the thermal preference of mice. This part of the study was based on the comparison of the time spent on the same plate before and after duloxetine, or pregabalin injection. It revealed that for the duloxetine-treated mice a statistically significant difference in time spent on the same plate after oxaliplatin (predrug) vs. after duloxetine (postdrug) administration was noted for temperatures: 0–5 °C (*p* < 0.001, Figure 1A), 5–10 °C (*p* < 0.0001, Figure 1B), 30–35 °C (*p* < 0.05, Figure 1G) and 40–45 °C (*p* < 0.0001, Figure 1G). This effect was particularly enhanced for temperatures 40–45 °C (Figure 1I).

For pregabalin-treated mice a statistically significant difference in time spent on the same plate after oxaliplatin (predrug) vs. after pregabalin (postdrug) treatment was noted for temperatures: 0–5 °C (*p* < 0.0001, Figure 1A), 5–10 °C (*p* < 0.0001, Figure 1B), 10–15 °C (*p* < 0.001, Figure 1C), 15–20 °C (*p* < 0.01, Figure 1D), 20–25 °C (*p* < 0.0001, Figure 1E) and 25–30 °C (*p* < 0.01, Figure 1F). Importantly, in contrast to duloxetine, in the experiment carried out at 40–45 °C, the effect of pregabalin on time spent on the same plate was marginal (Figure 1I).

Taken together, these data demonstrated that the loss of thermal preference or reversal of animal’s thermal preference towards a physiologically less comfortable temperature could be a measure of a drug’s analgesic action. We also showed that the analgesic action exerted by duloxetine was not detected at all temperature ranges tested. In other words, not all temperatures constitute optimal conditions for the detection of analgesic action of duloxetine.

Pregabalin influenced thermal preference of oxaliplatin-treated mice within the whole range of temperatures tested. Therefore, its potential analgesic action measured as the loss of thermal preference or its reversal towards a less comfortable temperature seems to be not as selective as that of duloxetine. It has to be emphasized here that on the one hand, for temperatures below thermal preferendum (i.e., below 25 °C) this activity of pregabalin can be considered a measure of the analgesic action. At temperatures exceeding thermal preferendum (above 35 °C), the observed preference of mice towards a zone with a lower temperature and avoidance of high temperatures, which are potentially harmful to the skin, may indicate undisturbed perception of thermal pain stimuli (i.e., heat). This, in turn, should be regarded as an important feature of a drug tested.

## 3. Discussion

Increased sensitivity to cold is a hallmark of oxaliplatin-induced neuropathy [1]. Preclinical methods widely used to assess cold-induced hypersensitivity in oxaliplatin-treated mice and rats comprise the cold plate test, cold water test and the acetone evaporation test [7]. These assays rely on applying a cold stimulus to a mouse’s or a rat’s paw or tail to measure pain-like responses (e.g., paw lifting, paw licking) [8]. Of note, these methods based on cold stimulation of neuropathic subjects use a constant temperature chosen from two temperature ranges to generate cold stimuli: 0–4 °C (mouse), or 5 °C (rat) to assess cold hyperalgesia, and alternatively, cooling (10 °C) to assess cold allodynia in mice and rats [7].

In the literature, there are very few preliminary studies that assessed thermal preference of rats [6] and mice [9]. The former study [6] was focused on monitoring time spent by unrestrained rodents on a two-plate device with one test plate and the other one—the reference plate, which was set at a constant, neutral temperature (25 °C). This study showed that thermal preferences of healthy rats were more than 17 °C and less than 40 °C. When compared with control (i.e., non-neuropathic) animals, oxaliplatin-treated neuropathic rats showed thermal hypersensitivity at 12, 20 and 35 °C.

In our present mouse study we used a distinct protocol to assess animals’ preference for various temperature ranges, i.e., we did not use a reference plate and both plates were randomly cooled or heated to obtain thermal zones adjusted every 5 °C on each plate. This experiment confirmed that, similar to rats, naïve mice preferred a warmer plate in the temperature setting range up to 30–35 °C. Additionally, neuropathic, oxaliplatin-treated mice, avoided a colder plate of the two used, and this preference was mainly seen up to 25–30 °C.

Both duloxetine [10] and pregabalin [11,12] are stable at the mouse’s body temperature and at room temperature but their analgesic activity in vivo assessed in response to various thermal stimuli is different. For instance, previous studies showed that duloxetine is effective in reducing cold-exacerbated pain measured in the cold plate test in oxaliplatin-treated mice [8], while pregabalin was only weakly effective in the mouse cold water test in CIPN models induced by paclitaxel or oxaliplatin [13] but it showed antiallodynic properties in the cold water test in a mouse model of painful diabetic neuropathy [14]. Additionally, many studies that used the hot plate test have demonstrated that duloxetine was effective in this assay [15,16], while the efficacy of pregabalin significantly depended on the model used [14,17,18]. Taken together, these studies showed that the analgesic activity of duloxetine and pregabalin were different, if mice were exposed to cold, or heat stimuli.

In the above mentioned rat study [6], duloxetine (2.5 mg/kg, intraperitoneal administration) reversed oxaliplatin-induced cold hypersensitivity at 20 °C. It was concluded that the two-plate method potentially enables the assessment of thermal sensitivity. This method seems to be useful and relevant to the pathophysiological exploration of animal pain models and to the assessment of analgesic efficacy of drugs and drug candidates but further, more detailed studies are needed to gain a deeper insight into phenomena underlying thermal preference of neuropathic animals and to assess how analgesics modulate these phenomena.

In our present research duloxetine was used at a higher, previously tested in the classic mouse cold plate test (2.5 °C), analgesic active dose, i.e., 30 mg/kg [8]. Its intraperitoneal administration to mice resulted in the abolition or inversion of thermal preference compared to that observed before duloxetine treatment. Hence, we concluded that the observed effect could be interpreted as a measure of this drug’s analgesic activity in the oxaliplatin-induced neuropathic pain model. Importantly, this effect was particularly enhanced at temperatures between 0 and 10 °C and it showed that duloxetine was able to attenuate cold-induced pain. At temperatures ranging between 15 and 30 °C duloxetine did not really work, which indicated that at these temperatures duloxetine had no effect on thermal nociceptive threshold in neuropathic mice. Taken together, this part of the present study also confirmed the validity of using temperatures up to 10 °C to assess the analgesic effect of drugs in the model of oxaliplatin-induced neuropathy (cold-induced pain).

In the experiment that was focused on the assessment of the activity of pregabalin, we used a fixed dose of this drug (30 mg/kg). This dose was previously tested in the oxaliplatin-induced neuropathic pain model and although it attenuated tactile allodynia in neuropathic mice, it did not reduce cold allodynia, or cold hyperalgesia [13]. The efficacy of pregabalin in reducing cold hypersensitivity is still a subject of debate and so far both preclinical and clinical studies have given many inconclusive data [1,19,20,21,22,23,24]. In our present study we observed that pregabalin-treated mice showed preference towards a colder plate of the two used and this effect was observed at temperatures 0–45 °C.

Importantly, both pregabalin and duloxetine in a similar way affected thermal sensation at temperatures above the thermal preferendum, i.e., both drugs maintained thermal preference towards a colder plate of the two used at temperatures exceeding 30 °C. This beneficial effect is potentially protective against body harm due to heat damage. Nociception is the perception of mechanical, thermal or chemical stimuli and its role is to protect body tissues against potential harm. This means that the restoration or maintenance of physiological pain thresholds enables one to avoid potential damage to the body. In our research, the observed preference of duloxetine- or pregabalin-treated mice towards thermal zones with temperatures closer to the thermal preferendum (i.e., the avoidance of temperatures higher than 30 °C) might indicate that at temperatures exceeding 30 °C the nociceptive thresholds were not elevated by both drugs far beyond baseline values of untreated control mice. At the same time, we showed that the method we propose, enabled to differentiate between a drug’s action within desired and undesired thermal ranges and this, in turn, might be helpful in selecting only those drug candidates whose pharmacological action will not be excessive, thus being responsible for a diminished protection against harmful thermal stimuli. Hence, this method will also enable us to assess possible adverse effects associated with an excessive therapeutic effect of drugs (induction of sensory disturbances and loss of physiological sensation—hypoalgesia). This will additionally allow one to select safe dose ranges of compounds and drugs that are able to maintain or restore physiological nociceptive threshold, and toxic doses, which should be avoided as potentially harmful.

Importantly, our research enabled to differentiate between the mode of analgesic action of the two drugs tested, i.e., duloxetine and pregabalin. Considering that cold hyperalgesia is a key symptom of oxaliplatin-induced CIPN, pain management strategies for this debilitating clinical condition should be focused on the attenuation of cold hypersensitivity, i.e., drugs should provide analgesia selectively at low temperatures without impairing nociception at temperatures within thermal preferendum for naïve (healthy) mice. Our study demonstrated that the potential analgesic action of duloxetine, measured as abolition or inversion of preference towards a warmer plate of the device, was rather observed at temperatures below thermal preferendum. In contrast to this, within temperatures optimal for healthy mice, duloxetine did not have such properties but at the same time, within this thermal range, the pain threshold of oxaliplatin-treated mice resembled that of naïve mice. Unlike duloxetine, pregabalin was not selective for temperatures below thermal preferendum and it influenced pain sensation at a much wider range of temperatures applied. So, considering this, for this particular type of neuropathy, duloxetine seems to be a better therapeutic option than pregabalin.

So far, many pharmacological strategies to treat cold-exacerbated pain have failed to provide analgesia in oxaliplatin-treated animals [1,13,22,25,26] and humans [1,26,27,28]. On the other hand, it is also likely that (1) the in vivo methods used at the preclinical stage (i.e., behavioral tests) might not be sensitive enough to detect analgesic active compounds, or (2) the parameters used to assess analgesic activity (i.e., temperatures at which chemical compounds were tested) are suboptimal. The above mentioned work by Balayssac and colleagues [6] has demonstrated that oxaliplatin-treated rats showed thermal hypersensitivity at 12, 20 and 35 °C, and the reference drug-duloxetine (2.5 mg/kg, intraperitoneal) reversed oxaliplatin-induced cold hypersensitivity at 20 °C. We showed that in mice the analgesic action of duloxetine is observed at a lower than 20 °C temperature range but importantly, the classic mouse cold plate or water immersion tests also use these low temperatures. Considering this, it can be concluded that these preclinical tests use temperatures optimal for the detection of analgesics effective in the mouse oxaliplatin model.

To sum up, in order to develop a novel, sensitive and accurate method to study thermal preference of oxaliplatin-treated neuropathic mice and to assess the activity of potential analgesics for this pain type, we undertook this research to compare thermal preference of mice at a broad range of temperatures (0–45 °C). The two reference analgesics that we used showed different selectivity in terms of their effect on the thermal pain threshold in neuropathic, oxaliplatin-treated mice.

Regarding the effects of duloxetine and pregabalin (or hypothetically any other analgesic drug for thermally-induced pain), first we expected that the analgesic effects of a drug that can attenuate cold-induced pain will be measured as more balanced time spent in both temperatures rather than preferring a colder temperature. However, after test drug administration we observed both a loss of preference to one of the plates, or thermal preference reversal. We considered several potential explanations for the observed reversal of the thermal preference of mice, including a drug’s specific mode of action. It is also likely that the observed effect of a fixed dose depends on the temperature range applied. This means that in certain temperature ranges, (e.g., 0–5 °C for both drugs), the drug dose used works in a more balanced manner and abolishes preference, while in other temperature ranges it causes the avoidance of a particular thermal zone and this results in the reversal of thermal preference. It should also be noted that the loss of preference observed after administration of duloxetine in the temperature range between 20 and 35 °C (and also no effect of duloxetine in 25–30 °C) may be related to the fact that this is the range of the physiological thermal preferendum, therefore each of the set temperatures was potentially comfortable for the animals.

We can also consider possible consequences of the reversal of the animals’ thermal preferences following the administration of analgesics. This could be not only an analgesic effect, but also an undesirable effect related to an excessive pharmacological effect of a drug. This effect appears to be potentially dangerous and is due to the impaired nociception (i.e., reduced sensation of harmful stimuli, which might lead to the paw tissue damage). Thus, the proposed here method allows not only to establish the effect of the drug and the dose used on the thermal pain threshold (assessment of the analgesic effect), but it may also be helpful to assess whether a given drug dose is potentially dangerous.

Our preliminary observations seem to be important also from the translational point of view. This means that the method might be useful in the search for effective and safe analgesics for reducing symptoms caused by thermal stimuli in the course of CIPN. In the context of the translational value, the method may be important because it enables a precise observation of animals’ behavior—classic methods for measuring pain induced by a thermal stimulus are based on the exposure of an animal to a thermal stimulus within a 5–10 times shorter testing period as compared to that which was used in our experiment. The sufficient 5-min observation time used in our research, and careful analysis of the collected images allow, in addition to examining the analgesic effect, to identify possible non-painful symptoms that appear in the course of CIPN (motor impairments, altered locomotor activity of mice, etc.). As mentioned above, the analysis of the time spent by the animal on one of the plates, measured as a significant increase of this parameter compared to that measured before administration of the test compound, additionally supported by the analysis of images and video recordings collected during the experiment, can significantly increase the reliability of the study (increased construct validity due to replication of more symptoms of the disease, increased face validity and predictive validity). Importantly, changes in the preference for one of the plates of the apparatus may sometimes indicate a sedative, or a stimulant effect of the test compound. At this point, it should be emphasized that in our present studies, neither duloxetine, nor pregabalin produced such false positive effects in any of the temperature ranges used.

Moreover, the proposed method offers more possibilities than the classic methods used in thermally-induced pain tests (hot plate test and cold plate test), because, unlike these methods using one rigidly set thermal point, it is able to test thermal preferences of animals in any (and not only a 5-degree) temperature range. Additionally, an advantage of the method in the context of the translational value of research is that it enables the assessment of the drug’s effect on the pain threshold under the conditions using two distinct thermal stimuli at the same time.

One of the limitations of our present research is that there are only few literature references to compare our data, which results in the inability to fully interpret the obtained preliminary observations. Most of the research in pain studies focuses on measuring the response of animals with the use of hot and cold plate tests. In our experiment, we used two different temperatures simultaneously. Based on the data from classic methods, we expected to observe cold-hypersensitivity of oxaliplatin-treated mice compared to the control groups (before oxaliplatin). This means that we assumed an increased amount of time spent on a warmer plate by mice that received oxaliplatin compared to control mice. Surprisingly, in our research, we did not observe such an effect, and at the moment the explanation for this is not entirely clear. In the case of the lower temperature ranges, it can be assumed that this indicates that both temperatures the mice (non-neuropathic and neuropathic) were exposed to were likely to be suboptimal, or even harmful and hence the animals searched for the optimal thermal zone throughout the experiment duration. In the higher temperature range, the plausible explanation for the observed lack of difference between these two measurements is perhaps that both temperatures used are equally comfortable. These presumptions point out for an urgent need to conduct further, extended research to assess animals’ thermal avoidance using devices and methods in which a mouse will be exposed to nine temperatures at one time. This will enable to measure not only thermal preference, but also the avoidance of harmful temperatures.

It should also be noted that the described here method was used in the oxaliplatin-induced CIPN model. In the future, the efficacy of the proposed method and the effects of drugs and analgesic drug candidates should be additionally assessed in other CIPN models (e.g., vincristine model, paclitaxel model, etc.), and in other than CIPN neuropathic pain models (e.g., chronic constriction injury model and streptozotocin-induced diabetic neuropathic pain model) because at present there is not any single animal model that is able to represent the full clinical situation. Therefore, interspecies differences and differences between the conditions created in animals and those observed in humans suffering from neuropathic pain symptoms should stimulate scientists to develop methods and animal models that would increase our confidence in the translational nature of research and its applicability for the clinical situation. Additionally, testing analgesics not only in male mice but also in female animals seems to be challenging.

## 4. Materials and Methods

### 4.1. Animals

In vivo experiments were performed at the Department of Pharmacodynamics, Faculty of Pharmacy, Jagiellonian University Medical College, Krakow. Behavioral tests were performed between 9 AM and 2 PM. The experimental procedure was approved by the 2nd Local Ethics Committee in Krakow. The treatment of animals was in full accordance with ethical standards laid down in respective Polish and EU regulations (Directive 2010/63/EU). The investigators involved in behavioral assays were blinded to experimental groups and this enabled us to avoid potential bias in data recording. Adult male Albino Swiss (CD-1) mice weighing 18–22 g were used in the experiment. They were purchased from the Animal Breeding Farm of the Faculty of Pharmacy, Jagiellonian University Medical College. Prior to in vivo tests, the mice were kept in standard laboratory conditions (groups of 10 mice in standard plastic cages, bedding material (Transwiór, Poland) at least 2 cm deep, room temperature of 22 ± 2 °C, light/dark (12:12) cycle, lights on at 8 AM, humidity 50% ± 10%, free access to tap water and food (Murigran, Agropol, Poland), environmental enrichment in cages). Experimental groups consisted of 8–10 randomly selected animals/dose. The mice were euthanized by cervical dislocation after completing the assay.

### 4.2. Chemicals

In the in vivo experiment duloxetine (donated from Adamed, Poland) and pregabalin (Tocris Bioscience, Germany) were used as suspensions prepared in 1% Tween 80 solution (Baxter, Poland). Analgesic drugs and vehicle were administered intraperitoneally 1 h before the measurements of their effect on pain threshold. To induce neuropathy, oxaliplatin (Activate Scientific GmbH, Germany) dissolved in 5% glucose solution (Polfa Kutno, Poland) was intraperitoneally injected at a single dose of 10 mg/kg. This dose was selected on the basis of our previous research [8,13] and available literature data [29,30]. Doses of duloxetine and pregabalin were selected based on our previous studies [8,13]. They showed that the dose 30 mg/kg of both drugs was optimal for studying their effect on the nociceptive threshold. The previously tested lower dose (10 mg/kg) was only marginally effective, while doses higher than 30 mg/kg were likely to give false positive results in pain tests due to increased sedation [31].

### 4.3. Equipment Used to Assess Thermal Preference in Mice

**Figure 2:**
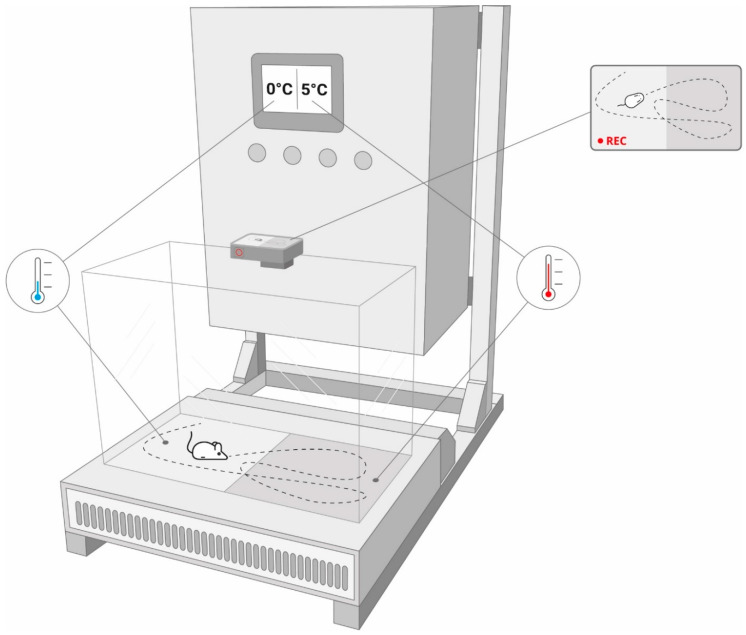
The prototype analgesiameter used in this study to assess thermal preference of naïve and neuropathic mice.

The two-plate analgesiameter for freely moving mice was used in this study (Figure 2). This prototype device is our own solution, which has several advantages as compared to classical analgesiameters, such as: (1) measuring only thermally-evoked pain, (2) controlling current temperature on each plate of the device using advanced proportional-integral-derivative (PID) algorithms implemented in PLC S7-1200 controller (Siemens, Germany) [32], which allows the mouse to choose the preferred thermal zone, without being forced by the researcher, (3) rapid and precise regulation of the temperature set-point on each of the two plates of this device enables to establish separate “thermal zones” randomly in each testing session, which significantly limits potential false positive results due to animals’ ability to learn choosing the preferred thermal zone of the device (i.e., a preferred plate) based on external visual cues, (4) the possibility of setting narrow-range thermal zones on each plate, which increases sensitivity of the method; at the same time this allows to measure the effect of a drug on hyperalgesia and allodynia, (5) the measurements are collected from unrestrained animals and therefore this method is less stressful for animals as compared to some standard thermal pain tests (e.g., cold water test and cold plate test), thus offering more reliable data due to a reduction of stress-related behavioral reflex responses of experimental animals (e.g., stress-induced analgesia) [33] and (6) higher objectivity of measurements (i.e., observer-independent assessment) because time spent by the animal in a given thermal zone and trajectories are recorded by the video camera (GoPro Hero7 Black, San Mateo, CA, USA) and subsequently they are analyzed with the use of mathematical methods of image analysis (deep learning and machine learning) [34,35]. Of note, the camera used for the detection of animals’ movements enables one to assess the effect of test drugs on locomotor activity of mice and this allows one to exclude potential false positive results in the pain test that are due to decreased locomotor activity [31].

Our own experience in pain research indicates that the assessment of a drug effect on thermally induced pain is difficult and requires the observer’s extensive experience. Available methods assessing pain-like behavior in rodents are highly subjective—they depend on the observer’s perception, so in order to obtain reliable data, it is of key importance that the measurements are made by the same experimenter blind to experimental conditions. Moreover, animal responses can result from the animal’s locomotor activity and can be misinterpreted. In addition to this, paw lift techniques examining heat or cold hypersensitivity (hot/cold plate apparatus) or assessing tactile allodynia (von Frey filaments) are not performed or scored similarly in various laboratories and a plethora of behavioral indices measured (e.g., total number of paw lifts, latency to the first paw lift) is striking. Moreover, in these techniques, it is uncertain, if a paw lift is only caused by thermal hypersensitivity or is due to spontaneous pain that might be enhanced during the test. Additionally, there may be intraindividual differences in scoring paw lifts by the experimenter, which is also a potential confounding factor in performing these assays. The presence of the experimenter in the experimental room might also have influence on the results obtained. Therefore, the proposed method here based on measuring the thermal preference of mice to select drugs for a particular pain condition seems to be more reliable than the available classical assays used for the detection of pain threshold in mice.

### 4.4. Assessment of Thermal Preference of Mice Exposed to Oxaliplatin

One day before the behavioral test, mice were habituated to the device and test conditions (room temperature: 22 ± 2 °C on both plates; habituation period: 5 min). On the test day, the thermal preference of mice was assessed at 3 time points, i.e., before oxaliplatin administration (referred to as “before oxaliplatin”), 3 h after oxaliplatin (“pre-drug”) and 1 h after duloxetine or pregabalin administration (“post-drug”). For this purpose each mouse was placed on a starting plate (i.e., in each experimental session a starting plate was a plate with a lower temperature set). Then, the mouse was allowed to explore both plates of the analgesiameter for 5 min. Time spent by the mouse in each thermal zone, i.e., on each plate of the device was recorded using a video camera (GoPro Hero7 Black, San Mateo, CA, USA) and analyzed using support vector classification (SVC) and deep learning methods. The test was performed in 9 separate sessions, i.e., at thermal zones of the two plates ranging between 0 and 45 °C, adjustable every 5 °C, i.e., 0–5, 5–10, 10–15, 15–20, 20–25, 25–30, 30–35, 35–40 and 40–45 °C (sessions 1, 2, 3, …, etc.).

### 4.5. Data Analysis

In vivo results were analyzed with the use of GraphPad Prism software (version 8.0, San Diego, CA, USA). The results obtained in behavioral tests are expressed as % time spent in each thermal zone. Statistical evaluation of the results was carried out using repeated measures ANOVA and Tukey’s multiple comparison for group comparisons made repeatedly at different time-points (i.e., before oxaliplatin, predrug and postdrug measurements).

The analysis of time spent in individual thermal zones was conducted using SVC and genetic algorithms, which enabled preparation of data from image analyses for subsequent statistical analyses. Preparation of algorithms for image analyses—here, classical methods of analysis supported by advanced machine learning-based methods (e.g., deep learning) were used.

Image analysis is based on the concept of background subtraction algorithm [36]. In each iteration the program determined whether the mouse was in the left or right part of the stand. The program captured two consecutive frames and performed image processing: resizing, grayscale conversion and median filtering. Then image subtracting followed. Afterwards, the resulting image was binarized and the median filtering was performed to remove noises. In the final stage, the algorithm counted the white pixels (i.e., with value equals one) separately in the left and right part of the image. A higher value indicated the area where mouse’s movement had been detected. The program was repeated until the end of the recording.

## Data Availability

The data presented in this study are available on request from the corresponding author. The data are not publicly available due to preparation for patent proceedings.

## Sample Availability

Samples of the compounds are not available from the authors.

## Abbreviations

ASCO	American Society of Clinical Oncology
CIPN	Chemotherapy-induced peripheral neuropathy
PID	Proportional-Integral-Derivative
SVC	Support Vector Classification
